# A Bioartificial Device for the Encapsulation of Pancreatic β-Cells Using a Semipermeable Biocompatible Porous Membrane

**DOI:** 10.3390/jcm14051631

**Published:** 2025-02-27

**Authors:** Nicola Cuscino, Salvatore Castelbuono, Claudio Centi, Rosaria Tinnirello, Maura Cimino, Giovanni Zito, Andrea Orlando, Massimo Pinzani, Pier Giulio Conaldi, Alessandro Mattina, Vitale Miceli

**Affiliations:** 1 Research Department, IRCCS ISMETT (Istituto Mediterraneo per i Trapianti e Terapie ad Alta Specializzazione), 90127 Palermo, Italy; ncuscino@ismett.edu (N.C.); scastelbuono@ismett.edu (S.C.); ccenti@ismett.edu (C.C.); rtinnirello@ismett.edu (R.T.); mcimino@ismett.edu (M.C.); gzito@ismett.edu (G.Z.); aorlando@ismett.edu (A.O.); mpinzani@ismett.edu (M.P.); pgconaldi@ismett.edu (P.G.C.); 2 Department of Engineering, University of Palermo, 90128 Palermo, Italy; 3 Department of Biomedicine, Neurosciences and Advanced Diagnostics (BiND), University of Palermo, 90127 Palermo, Italy; 4 Diabetes Service, IRCCS ISMETT (Istituto Mediterraneo per i Trapianti e Terapie ad Alta Specializzazione), 90127 Palermo, Italy; amattina@ismett.edu

**Keywords:** type 1 diabetes, β-cell encapsulation, pancreatic islets, immunoisolation device, cell transplantation

## Abstract

**Background/Objectives**: Type 1 diabetes (T1D) is a chronic autoimmune condition characterized by the destruction of pancreatic β-cells, leading to insulin deficiency. Current therapies, such as islet transplantation, face significant challenges, including limited donor availability and the need for lifelong immunosuppression. Encapsulation technologies offer a promising alternative, providing immune protection and maintaining β-cell viability. In this study, we propose an encapsulation device featuring a spiral tubular semipermeable polyethersulfone (PES) membrane reinforced with a rigid biocompatible resin scaffold. **Methods**: The PES membrane was engineered with a tailored porosity of 0.5 µm, enabling efficient nutrient and oxygen exchange while preventing immune cell infiltration. Using INS-1E insulin-secreting cells aggregated into size-controlled islet-like spheroids (ILSs), we evaluated the device’s performance. **Results**: The device achieved high ILS viability and insulin secretion over 48 h at therapeutic densities, maintaining functionality comparable to free-floating ILSs (control). The PES membrane, with its mechanical stability and biocompatibility, ensured durability without compromising diffusion dynamics, overcoming a critical limitation of other encapsulation approaches. Importantly, the device geometry allowed for the encapsulation of up to 356,000 islet equivalents (IEQs) in a single capillary fiber, reaching therapeutic thresholds for T1D patients. **Conclusions**: this device, with its innovative design, enables high-density encapsulation while preserving ILS functionality and scalability, making it a potential platform for clinical application. This work highlights the potential of PES-based encapsulation devices to overcome key barriers in T1D treatment, paving the way for personalized, long-term solutions to restore insulin independence.

## 1. Introduction

Type 1 diabetes (T1D) is a severe autoimmune disorder characterized by the selective destruction of insulin-producing β-cells within the islets of Langerhans, resulting in a severe deficiency of insulin production [1]. This condition primarily affects children and young adults, with the overall global prevalence of T1D increasing from 2,376,444 in 1990 to 3,644,613 in 2019, showing a 53.36% increase over the 30-year period [2,3]. In 2022, the global prevalence of type 1 diabetes was estimated at 8.75 million individuals, of whom 1.52 million were under the age of 20 [4]. Current treatment strategies for T1D include exogenous insulin injections. However, insulin therapy often falls short of achieving the precise glycemic control exhibited by a healthy physiological system, leading to hypoglycemic events and long-term complications in many patients [5,6]. β-Cell replacement therapies, such as pancreas or islet transplantation (ITx), are instead considered a promising curative option [7,8]. Islet transplantation, in particular, has emerged as a promising approach for restoring insulin independence in T1D patients. However, the clinical success of ITx is hampered by a shortage of donor cells, as the availability of high-quality pancreas for islet isolation is extremely limited [9,10]. Furthermore, ITx typically requires lifelong immunosuppressive therapy to prevent graft rejection, which increases the patient’s risk of infections and certain types of cancer [9,11]. Consequently, innovative strategies are urgently needed to improve the survival and function of transplanted β-cells while minimizing the requirement for immunosuppression.

Various encapsulation strategies, including macroencapsulation and microencapsulation, have been developed to address the challenges of cell survival and functionality post-transplantation. For instance, some macroencapsulation methods with biocompatible polymeric macrocapsules involve the use of larger devices that physically contain the islets and provide them with a controlled environment [12]. This approach can, in some cases, facilitate vascularization within the device and the retrieval of encapsulated islets for analysis or replacement, thus allowing for personalized treatment protocols. However, the larger pore sizes in these macroencapsulation devices often permit immune cell penetration, necessitating the continued use of immunosuppressive drugs. In contrast, microencapsulation systems aim to create smaller, functional microcapsules that can enhance the viability of encapsulated β-cells by minimizing the transplantation volume while maximizing cell protection [13]. Although microencapsulation techniques have shown promise, many current hydrogel-based microcapsules exhibit limitations in cell viability due to restricted intercellular communication and nutrient diffusion [14]. As a result, developing an immunoprotective microencapsulation device that maintains optimal β-cell function and longevity in vivo remains a significant challenge. Recent advancements in the design of microencapsulation devices have led to innovative structures capable of providing both biocompatibility and adequate permeability for nutrient exchange [15]. A wide variety of materials have been used to synthesize these devices, including alginate, polyethylene glycol (PEG), polytetrafluoroethylene (PTFE), and polyethersulfone (PES) [16,17]. The latter is widely used in cell transplantation due to its mechanical properties, versatility, and high biocompatibility [18,19]

In this study, we propose an encapsulation device consisting of a spiral tubular arrangement of a semipermeable PES membrane enclosed within a rigid biocompatible resin scaffold. This design allows β-cells to be seeded while ensuring that they can interface with the external environment through tailored porous membranes. The porosity of this device is carefully engineered to facilitate the inflow of essential nutrients and oxygen while simultaneously shielding the β-cells from immune attack.

While ITx offers a potential way to restore insulin independence in T1D patients, significant challenges remain regarding donor cell scarcity and the need for immunosuppression. The development of effective encapsulation strategies holds great promise for enhancing the viability and longevity of either islets or stem cell-derived β-cells. In particular, stem cell-derived β-cells have emerged as a promising avenue for providing an unlimited source of insulin-producing cells for diabetes treatment. Recent studies have demonstrated the potential of these cells to exert glucose control in diabetic models [20]. Therefore, the introduction of innovative encapsulation strategies, such as the proposed PES-based encapsulation device, may represent a significant step forward in personalizing the treatment of T1D, ultimately improving patient outcomes and quality of life.

## 2. Materials and Methods

### 2.1. Adherent Cell Culture

Rat pancreatic INS-1E β-cells were grown in RPMI 1640 medium (Thermo Fisher Scientific, Waltham, MA, USA) supplemented with 10% fetal bovine serum (Thermo Fisher Scientific), 10 mmol/L HEPES, 1 mM sodium pyruvate, 2 mmol/L L-glutamine, 50 µM 2-mercaptoethanol, 100 U/mL penicillin, and 100 mg/mL streptomycin (Thermo Fisher Scientific) at 37 °C with 5% CO_2_. INS-1E cells were seeded in tissue culture-treated polystyrene flasks at a density of 1.5 × 10^4^ cells/cm^2^, and the medium was changed every 48 h. Cells were passaged at 90% confluency using 1× trypsin EDTA (Thermo Fisher Scientific).

### 2.2. Generation of INS-1E Islet-like Spheroids (ILSs)

INS-1E cells were seeded in 24-well spherical plates containing 750 microwells per well (Kugelmeiers, Erlenbach, Switzerland) to generate islet-like spheroids (ILSs). To obtain ILSs with a diameter of approximately 200 µm, we seeded INS-1E cells at a density of 7.5 × 10^5^ cells per well (1 × 10^3^ cells per microwell and per ILS). Cells were cultured for 72 h in complete medium supplemented with 5% fetal bovine serum at 37 °C with 5% CO_2_. Once the ILSs had formed, they were transferred to a new Ultra-Low Attachment plate (Corning, Corning, NY, USA) for one day of culture before being grown for 48 h either free-floating (200 ILSs) or in the encapsulation device (about 150,000 ILSs) in complete medium supplemented with 5% fetal bovine serum at 37 °C with 5% CO_2_.

### 2.3. Device Fabrication and Encapsulation of ILSs

**Figure 1:**
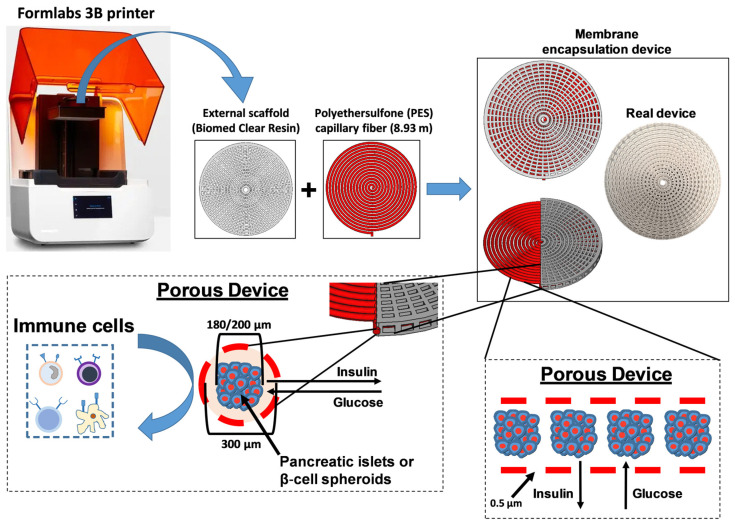
Schematic overview of the encapsulation device. The 3D scaffold was fabricated using BioMed Clear Resin and a Formlabs 3B printer. The scaffold was then integrated with a polyethersulfone (PES) capillary fiber, into which pancreatic islets or β-cell spheroids could be loaded.

First, we fabricated a 3D scaffold using BioMed Clear Resin with a Formlabs 3B printer (Formlabs, Somerville, MA, USA). In particular, the layer height was set to 100 μm and the resin temperature to 35 °C. Post-curing was essential for achieving the desired mechanical properties and biocompatibility. The printed parts were first rinsed with 99% isopropyl alcohol (IPA) for 20 min to remove the uncured resin and then dried. The final post-curing step involved heating to 60 °C for 60 min to optimize strength and clarity. The scaffolds were sterilized by autoclaving at 121 °C for 30 min prior to use. Next, we integrated the scaffold with a hydrophilic MicroPES Type TF10 hollow capillary fiber (Membrana, Charlotte, NC, USA), measuring 8.93 m in length and 300 µm in diameter (±40 µm) (Figure 1). This fiber features a semipermeable membrane, allowing the passage of proteins with molecular weights up to 400,000 Da. The membrane composition includes biocompatible PES with polyvinylpyrrolidone (PVP) added to optimize wettability and biocompatibility (Membrana). Subsequently, the fiber was attached to a Luer lock connector via a silicone bridge (Dow Corning, Midland, MI, USA), allowing the ILS suspension to be loaded into a cylindrical perfusion device via a syringe. Once the ILSs were loaded, the Luer lock connector was removed, and the fiber was sealed with silicone.

### 2.4. Viability Assays

To assess cell viability, we quantified cellular adenosine triphosphate (ATP) levels in ILSs grown either within the device or as free-floating spheroids for 24 and 48 h using the CellTiter-Glo 3D assay (Promega, Madison, WI, USA). For free-floating ILSs, 300 µL of CellTiter-Glo reagent was added to each well containing 300 µL of medium in a 24-well plate. For ILSs cultured within the device, the capillary fiber was carefully removed from the scaffold and transferred to a well of a 24-well plate. Each fiber was then treated with 600 µL of CellTiter-Glo reagent to lyse the ILSs, which released ATP. All samples were incubated at room temperature for 25 min to ensure complete cell lysis and stabilization of the luminescent signal. Given the considerable difference in cell number between device-grown and free-floating ILSs, the CellTiter-Glo reagent from device-grown ILSs was diluted 1000-fold prior to luminescence measurements on the Spark multimode microplate reader (Tecan, Mannerford, Switzerland) to allow for a comparable luminescence signal. Cellular ATP levels were normalized to the cell number for each experimental condition. The data were obtained from three independent experiments, each conducted in triplicate.

### 2.5. Evaluation of Membrane Transport Properties by Glucose-Stimulated Insulin Secretion (GSIS)

Adherent cells and ILSs grown within the device or as free-floating spheroids for 24 and 48 h were washed with PBS and pre-incubated for 30 min at 37 °C in glucose-free Krebs buffer supplemented with 0.1% BSA and containing (in mM) 125 NaCl, 5.9 KCl, 1.2 MgCl_2_, 1.3 CaCl_2_, and 25 HEPES, adjusted to pH 7.4 with NaOH. The glucose-free buffer was then replaced with the same buffer supplemented with either 4 or 15 mM glucose, and cells were incubated for 60 min at 37 °C. Supernatants containing released insulin were collected and stored at −20 °C until analysis, which was performed using an enzyme-linked immunoassay (Mercodia, Uppsala, Sweden) by following the manufacturer’s instructions. To normalize GSIS based on the cell number, adherent cells were counted after each experiment, while the initial cell count per spheroid was used to estimate total cell number, assuming that each ILS contained 1000 cells. The stimulation index (SI) was calculated as a ratio of the normalized insulin secreted in the high-glucose group to that of the low-glucose group.

### 2.6. Statistics

All data are representative of three independent experiments and are expressed as the means ± standard deviations (SDs). Data from different groups were compared using computerized statistical software with ANOVA. When ANOVA revealed *p* < 0.05, the data were further analyzed with Student’s *t*-test. Differences were considered statistically significant at *p* < 0.05.

## 3. Results

### 3.1. Fabrication and Characterization of the Encapsulation Device

To stabilize the device structure, we used a 3D printing process to produce an external scaffold with a defined shape and dimensions for the encapsulation device (Figure 1). The resulting scaffold had a radius of 31.5 mm and a height of 4 mm, featuring a polar pattern with 50 rectangular slots across 10 external concentric circles and 25 slots on 4 inner circles, spaced 1 mm apart. In Figure 1, we also show the second component of the device, a preconfigured tubular structure (encapsulation fiber) composed of a semipermeable PES membrane with an internal diameter of 300 µm, designed to encapsulate either ILSs or pancreatic islets with diameters of 150–200 µm. This PES membrane was specifically engineered with standardized porosity (0.5 µm) to allow the passage of molecules up to 400,000 Da, such as insulin and glucose, while effectively isolating the encapsulated β-cells from immune cells (Figure 1). Furthermore, the addition of PVP to the PES membrane enhanced hydrophilicity and prevented ILS adhesion to the membrane surface. Following device assembly, ILSs were loaded into the encapsulation fiber. Given the fiber length of 8.93 m and internal diameter of 300 µm, resulting in an internal volume of 630.5 mm^3^, we estimated that the encapsulation fiber could accommodate approximately 150,000 ILSs with a diameter of 200 µm (each ILS volume = 0.00419 mm^3^).

### 3.2. Production and Size Distribution of INS-1E Islet-like Spheroids (ILSs)

**Figure 2:**
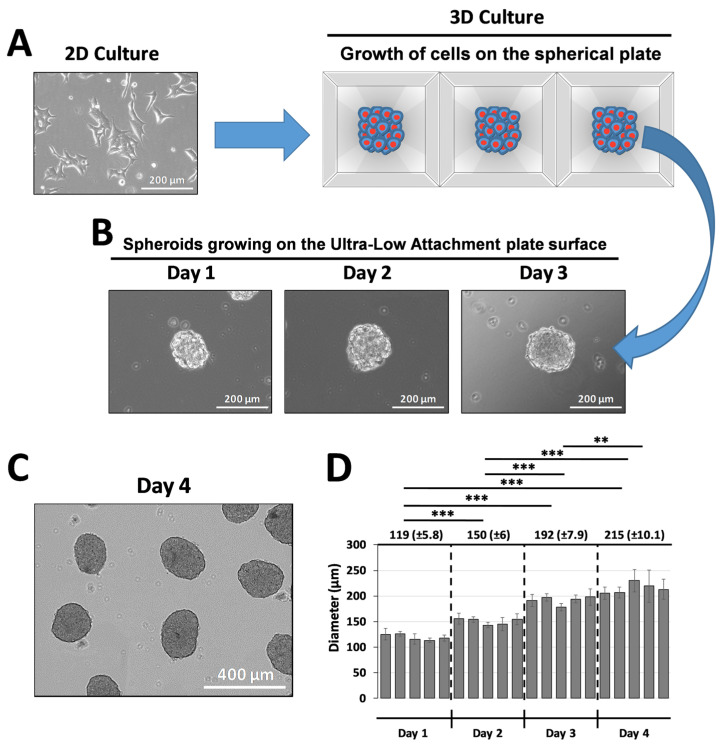
Generation of islet-like spheroids (ILSs). (**A**) Bright-field microscopy images of rat pancreatic INS-1E β-cells cultured in a monolayer and ILSs generated in the spherical plate. (**B**) Bright-field microscopy images of ILSs seeded in an Ultra-Low Attachment plate after formation in a spherical plate for 1, 2, and 3 days. (**C**) Bright-field microscopy images of ILSs cultured on an Ultra-Low Attachment plate for 1 day after formation in a spherical plate for 3 days. (**D**) Diameter distribution of ILSs. The data are presented as the means ± SDs of triplicate samples in three independent experiments. ** *p* < 0.01 and *** *p* < 0.001.

To determine the optimal conditions to generate homogeneous ILSs with a target diameter of approximately 200 µm (suitable for infusion into a capillary fiber with an internal diameter of 300 µm), INS-1E cells from 2D culture were initially seeded at various densities (500, 1000, and 2000 cells per microwell) in 24-well spherical plates (Figure 2A). We observed that seeding 1000 cells per microwell yielded compact ILSs of approximately 200 µm after 3 days of culture in the spherical plate (192 ± 7.9 µm) and an additional day of culture in an Ultra-Low Attachment plate (215 ± 10.1 µm) (Figure 2B,D). As shown in Figure 2C, after growing the ILSs in the spherical plate for 3 days, we performed an additional day of culture in an Ultra-Low Attachment plate to allow the ILSs to form an oval shape that more closely resembles the morphology of pancreatic islets in vivo.

### 3.3. Survival of ILSs

**Figure 3:**
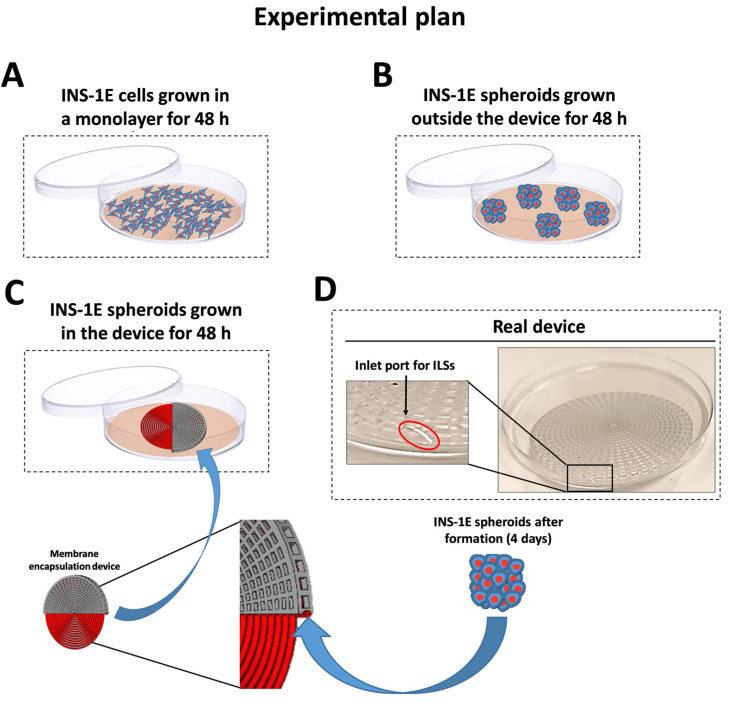
Detailed overview of the experimental plan for the cultivation and encapsulation of INS-1E cells. (**A**) INS-1E cells grown in monolayer culture. (**B**) Growth of INS-1E spheroids outside the encapsulation device after 48 h. (**C**) Growth of INS-1E spheroids within the encapsulation device. (**D**) Image of the real encapsulation device showing the inlet port for loading INS-1E spheroids into the device.

**Figure 4:**
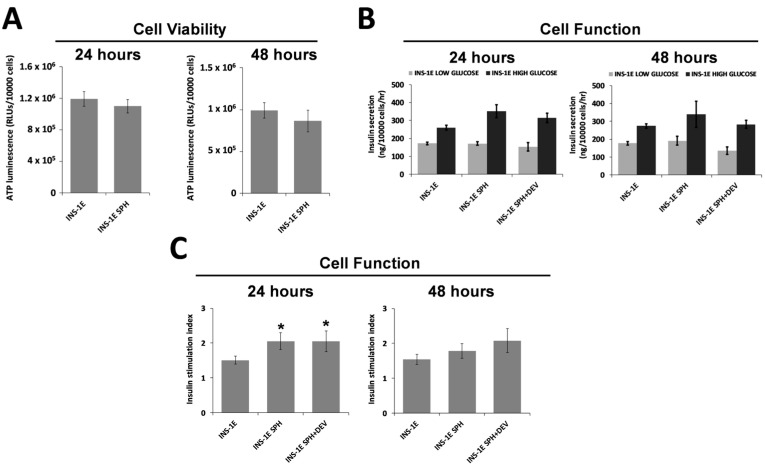
Assessments of the viability and functionality of encapsulated and non-encapsulated β-cells. (**A**) Cell viability, (**B**) cell function in terms of insulin release, and (**C**) cell function based on the stimulation index in INS-1E cells and INS-1E spheroids after 24 and 48 h of culture. INS-1E, INS-1E cells grown in monolayer; INS-1E SPH, INS-1E cells grown as spheroids outside the encapsulation device; INS-1E SPH+DEV, INS-1E cells grown as spheroids within the encapsulation device. The data are presented as the means ± SDs of triplicate samples in three independent experiments. * *p* < 0.05 versus INS-1E cells grown in a monolayer.

The primary requirement for β-cell encapsulation devices is to support islet encapsulation whilst maintaining viability. Therefore, the biocompatibility of the encapsulation fiber was assessed to evaluate its potential for biomedical applications. ILS viability was measured under both encapsulated and non-encapsulated conditions. Specifically, as shown in Figure 3, β-cell monolayer cultures were prepared (Figure 3A) or, following ILS formation in spherical plates, free-floating cultures (outside the device) (Figure 3B) or ILSs cultured within the encapsulation device (Figure 3C) were grown for 2 days. ATP levels, indicated by luminescence, were quantified based on the cell count per well. The results showed that no significant difference in viability was observed between free-floating ILSs and those cultured within the device after both 24 and 48 h (Figure 4A). These findings demonstrate the high biocompatibility of the encapsulation device.

### 3.4. ILS Functionality

Another essential feature of a β-cell encapsulation device is high permeability for insulin and glucose. To assess this, INS-1E cells cultured as either monolayers or ILSs (Figure 3) were exposed to low (4 mM) and high (15 mM) glucose concentrations. No significant differences in both glucose-stimulated insulin secretion (GSIS) (Figure 4B) and SI (Figure 4C) were observed between encapsulated and free-floating ILSs after both 24 and 48 h. These findings suggest that glucose effectively diffuses through the membrane into the inner compartment, stimulating insulin secretion from the ILSs, after which the insulin diffuses out of the device. Thus, the porosity of the microwell membranes appears sufficient to permit high levels of insulin and glucose transport without diffusion limitations.

## 4. Discussion

To address the persistent gap in the translation of β-cell encapsulation therapies for T1D from laboratory research to clinical applications, the design of effective encapsulation devices that overcome both islet survival and diffusion limitations is crucial [17].

In recent years, several macroencapsulation devices have been developed to protect transplanted β-cells from immune system attack. One of the first devices to be studied was TheraCyte, which incorporates a polytetrafluoroethylene (PTFE) membrane that serves as a physical barrier to immune cells while allowing the diffusion of nutrients and oxygen [21]. Preclinical studies have shown that this technology promotes neovascularization around the device, thereby improving graft survival. However, in most cases, the formation of a fibrotic capsule surrounding the device has been shown to compromise the functionality of the encapsulated islets. Among stem cell-based approaches, ViaCyte has developed two distinct Encaptra devices, VC-01 and VC-02, both designed with PTFE membranes for the transplantation of PEC-01 stem cell-derived pancreatic progenitor cells. VC-01 was engineered to provide complete immune protection without the need for immunosuppression. However, the clinical results revealed a strong fibrotic response, which limited its efficacy and ultimately led to its discontinuation [NCT02239354] [22]. VC-02, on the other hand, is a partially open device that enables direct vascularization of the implanted cells and, as a result, requires immunosuppression to prevent rejection [20]. In recent years, Vertex Pharmaceuticals (which has acquired ViaCyte) has been developing a fully encapsulated version of its stem cell-derived cell line, VX-264, designed to protect the implanted cells without the need for immunosuppression. However, clinical data are still limited [NCT05791201].

In our study, we demonstrate the feasibility of encapsulating β-cell-derived ILSs within a membrane-based device that optimizes nutrient and gas exchange. ILSs were seeded at a high density and had the opportunity to interface with the external environment to exchange nutrients, metabolites, and oxygen (Figure 1). This in vitro model provides an early-stage platform for assessing islet survival and function, the encapsulation architecture, and device scaling. Our device features a semipermeable membrane composed of PES surrounded by a rigid scaffold of biocompatible resin in a circular configuration (Figure 1). Emerging biomaterials such as zwitterionic hydrogels and modified alginates have shown promise in enhancing immune evasion and oxygen permeability [23]. However, PES remains a strong candidate for β-cell encapsulation due to its mechanical durability, chemical stability, tunable porosity, and established medical use [18,19,24,25]. The resin scaffold was fabricated using 3D bioprinting, enabling precise structural control and adaptability for implantation at various anatomical sites. Fabrication parameters, including the pore size and scaffold–membrane interconnectivity, were optimized to facilitate nutrient transport and maintain ILS viability at high densities. The key to our design was the integration of a semipermeable PES membrane with a tailored porosity of approximately 0.5 µm, which effectively prevents immune cell infiltration while allowing adequate nutrient and oxygen exchange. In fact, our results show that the tailored membrane porosity supports high levels of insulin and glucose transport, preventing any significant diffusion limitations. The level of porosity in our device strikes a balance between immune isolation and metabolic support, differentiating our approach from other polymeric macrocapsules that often utilize large pores, compromising immunoprotection [12]. Moreover, the biocompatibility of PES, combined with its mechanical resilience, offers the potential for long-term use without degradation [25], a necessary attribute for any clinical encapsulation device.

We assessed the device’s functionality using INS-1E insulin-secreting cells, which aggregate to form ILSs that closely mimic the metabolic characteristics of native pancreatic islets [26]. INS-1E cells offer a robust in vitro model that is frequently used in diabetes research [27,28] and for testing the efficacy of encapsulation technologies [24]. In our experiments, INS-1E cells were cultured in microwell plates to produce size-controlled, homogeneous ILSs with an average diameter of 213 µm (SD ±13.8 µm), aligning closely with our target range of 150–200 µm (Figure 2). This range was chosen to approximate the diameter of a functional islet equivalent (IEQ) of 150 µm, optimizing nutrient and oxygen diffusion [29]. These ILSs were easily loaded into the device due to an added practical advantage of our design, a small inlet port that simplifies seeding (Figure 3D), enabling rapid device preparation while reducing handling stress on the cells. We seeded a high density of ILSs within our device, reflecting the therapeutic requirements and achieving functional cell densities crucial for clinical applications. Our results demonstrate that ILSs encapsulated at high densities retained similar viability and insulin secretion function over 24 and 48 h compared to free-floating controls. In fact, the experimental results in Figure 4 show that ILSs survived and functioned both after 24 and 48 h when seeded at high numbers in the 300 µm thick capillary fiber. These effects might be ascribed to the fact that ILSs are in close contact with the external environment. Moreover, these findings align with the previous literature, suggesting that when the diffusion distance remains below 200 µm, cell survival and function are significantly improved, particularly at the higher densities essential for T1D treatment [14,30]. According to Dulong’s studies, to increase the number of functional islets, using a higher islet density in an implantable device is necessary to treat T1D [31]. The design geometry of our device proved essential in achieving a scalable encapsulation system capable of delivering a therapeutic dose of ILSs. With an inner diameter of 300 µm (±40 µm), our device can house a substantial volume of ILSs without compromising nutrient transport. Given that the Edmonton protocol defines a therapeutic dose as approximately 10,000 IEQs per kilogram of patient body weight, but only 3000–5000 IEQ per kg remain viable following intra-portal transplantation [32], our encapsulation system was scaled to address this requirement. Our calculations indicate that our device can accommodate up to 356,000 IEQs, exceeding the necessary therapeutic dose for a 60 kg individual. This capacity was achieved in a single capillary fiber of 8.93 m in length and 300 µm in inner diameter, which provides a volume of 630.5 mm^3^ and the opportunity of seeding up to 356,000 IEQs, which are 150 µm in diameter and occupy a volume of 0.00177 mm^3^. This calculation underpins a major challenge in the delivery of a therapeutic dose of islets in encapsulation devices and highlights the device’s potential to deliver a clinically relevant islet number. These conclusions highlight the device’s potential scalability while acknowledging that further validation is required.

Furthermore, findings in the literature, such as those of Lehmann et al., underscore the enhanced survival and insulin secretion capacity of smaller islets (diameter 150–200 µm) over larger islets, primarily due to superior mass transport properties [33]. Our encapsulation approach aligns with these findings, as we achieved high viability and functionality of ILSs, likely due to their favorable diameter range. Larger islets, which are often prone to necrosis due to limited nutrient diffusion [33], could thus be effectively replaced by smaller ILSs or pseudo-islets derived from stem cells in our device, potentially increasing the survival and functional yield of the encapsulated cells.

This study offers valuable insights into the development of a bioartificial device for the encapsulation of pancreatic β-cells. However, there are several limitations. The experiments were conducted exclusively in vitro, lacking the complexity of in vivo conditions, such as immune responses and the risk of fibrosis. The use of INS-1E insulin-secreting cells as a model, while reliable, may not fully represent the behavior of primary human islets or stem cell-derived β-cells. Additionally, the device’s ability to sustain oxygen and nutrient diffusion at high cell densities over extended periods is uncertain. Although its geometry supports high-density encapsulation, further research is needed to explore the scalability of this approach for clinical use, including implantation and retrieval. These limitations highlight the need for further preclinical and long-term in vivo studies to assess the clinical potential of this technology for treating T1D.

To address these challenges, our ongoing research focuses on refining the device design to improve its adaptability for in vivo studies. Furthermore, we are actively working on comparing the performance of our device to existing encapsulation systems to better understand its relative advantages and limitations.

## 5. Conclusions

This PES-based encapsulation device represents an early step toward developing a scalable bioartificial pancreas. By enabling the high-density encapsulation of β-cell-derived ILSs while maintaining their viability and function, this approach has the potential to support personalized treatment strategies for T1D. As advancements in β-cell sourcing, such as stem cell-derived β-cells, continue to progress, our device could serve as a versatile platform for future therapeutic applications. However, we emphasize that its clinical applicability remains preliminary, and further preclinical and long-term in vivo studies are essential to validate its translational potential.

## Data Availability

The original contributions presented in this study are included in the article. Further inquiries can be directed to the corresponding author.

## Abbreviations

ATP	Adenosine triphosphate
GSIS	Glucose-stimulated insulin secretion
HEPES	4-(2-Hydroxyethyl)-1-piperazineethanesulfonic acid
IEQ	Islet equivalent
ILS	Islet-like spheroid
INS-1E	Insulinoma-derived β-cell line (specific β-cell type used in research)
ITx	Islet transplantation
PES	Polyethersulfone
PVP	Polyvinylpyrrolidone
SI	Stimulation index
T1D	Type 1 diabetes
